# The CeHRes Roadmap 2.0: Update of a Holistic Framework for Development, Implementation, and Evaluation of eHealth Technologies

**DOI:** 10.2196/59601

**Published:** 2025-01-13

**Authors:** Hanneke Kip, Nienke Beerlage-de Jong, Lisette J E W C van Gemert-Pijnen, Saskia M Kelders

**Affiliations:** 1 Section of Psychology, Health & Technology Centre for eHealth and Wellbeing University of Twente Enschede Netherlands; 2 Department of Research Transfore Deventer Netherlands; 3 Optentia Research Unit North-West University Potchefstroom South Africa

**Keywords:** eHealth development, eHealth implementation, CeHRes Roadmap, participatory development, human-centered design, persuasive design, eHealth framework

## Abstract

To ensure that an eHealth technology fits with its intended users, other stakeholders, and the context within which it will be used, thorough development, implementation, and evaluation processes are necessary. The CeHRes (Centre for eHealth and Wellbeing Research) Roadmap is a framework that can help shape these processes. While it has been successfully used in research and practice, new developments and insights have arisen since the Roadmap’s first publication in 2011, not only within the domain of eHealth but also within the different disciplines in which the Roadmap is grounded. Because of these new developments and insights, a revision of the Roadmap was imperative. This paper aims to present the updated pillars and phases of the CeHRes Roadmap 2.0. The Roadmap was updated based on four types of sources: (1) experiences with its application in research; (2) literature reviews on eHealth development, implementation, and evaluation; (3) discussions with eHealth researchers; and (4) new insights and updates from relevant frameworks and theories. The updated pillars state that eHealth development, implementation, and evaluation (1) are ongoing and intertwined processes; (2) have a holistic approach in which context, people, and technology are intertwined; (3) consist of continuous evaluation cycles; (4) require active stakeholder involvement from the start; and (5) are based on interdisciplinary collaboration. The CeHRes Roadmap 2.0 consists of 5 interrelated phases, of which the first is the contextual inquiry, in which an overview of the involved stakeholders, the current situation, and points of improvement is created. The findings from the contextual inquiry are specified in the value specification, in which the foundation for the to-be-developed eHealth technology is created by formulating values and requirements, preliminarily selecting behavior change techniques and persuasive features, and initiating a business model. In the Design phase, the requirements are translated into several lo-fi and hi-fi prototypes that are iteratively tested with end users and other stakeholders. A version of the technology is rolled out in the Operationalization phase, using the business model and an implementation plan. In the Summative Evaluation phase, the impact, uptake, and working mechanisms are evaluated using a multimethod approach. All phases are interrelated by continuous formative evaluation cycles that ensure coherence between outcomes of phases and alignment with stakeholder needs. While the CeHRes Roadmap 2.0 consists of the same phases as the first version, the objectives and pillars have been updated and adapted, reflecting the increased emphasis on behavior change, implementation, and evaluation as a process. There is a need for more empirical studies that apply and reflect on the CeHRes Roadmap 2.0 to provide points of improvement because just as with any eHealth technology, the Roadmap has to be constantly improved based on the input of its users.

## Introduction

eHealth refers to the use of information and communication technology to support health, well-being, and health care [1]. If used well, eHealth technologies such as internet-based interventions, virtual reality, wearables, and mobile apps can be used to improve the quality and efficiency of care [2-6]. By achieving the intended effects, they can be used to target global challenges for health care, for example, limited access to care, an increase in the number of people living with (chronic) diseases, and shortages of staff and funding [7,8]. Over the past years, much attention has been paid to improving the impact and uptake of eHealth by academia, health care, and commercial companies. Yet, there still is room for improvement as expected benefits are often not (fully) achieved in practice [9,10].

An explanation for eHealth interventions not reaching their full potential is a suboptimal fit between the design of the eHealth technology, the characteristics and needs of its users, and the demands of the context in which it is used [11]. Therefore, thorough development, implementation, and evaluation processes are recommended to increase the fit between technology, people, and context and thus to realize the full potential of eHealth [12]. Unfortunately, this does not always happen. For example, despite the importance of participatory development for creating eHealth technologies, end users and other stakeholders are often not involved at all or not in the most optimal way [9,13]. Furthermore, during eHealth development, sufficient attention is not paid to behavior changes by integrating evidence-based behavior change theories [12,13]. In addition, the implementation of eHealth is a major challenge but often receives insufficient attention in research and practice [14,15]. Implementation is often seen as a single, postdesign activity, as opposed to an ongoing, iterative process that involves complex ecosystems and starts with the development of a technology. In addition, because eHealth technologies are quite new, there is an urgent need for more insight into if, why, how, when, and for whom they work, using innovative research methods that are suitable for answering these types of complex questions [16,17]. Finally, in research and practice, development, implementation, and evaluation projects are often conducted separately from each other, overlooking their interrelationships. Consequently, bolstering the impact of eHealth requires the use of comprehensive frameworks that guide interrelated development, implementation, and evaluation processes [12,18].

The CeHRes (Centre for eHealth and Wellbeing Research) Roadmap 2.0 is one such holistic framework and is specifically aimed at developing, implementing, and evaluating eHealth technologies that fit the needs and characteristics of people and their contexts. The original CeHRes Roadmap was first published in 2011 and is based on a comprehensive systematic literature search, resulting in 60 papers and 16 frameworks (van Gemert-Pijnen et al [11] provide a complete overview of the search process and outcomes). It included frameworks focused on, for example, user-centered design of teleconsulting systems [19], continuous evaluation of eHealth [20], usability engineering [21], interdisciplinary design and evaluation [22], and implementation and behavioral science [23]. These frameworks’ target groups, goals, foundations, and strategies and principles can be found in the study by van Gemert-Pijnen et al [11]. An analysis of the strategies and principles was used to create the original CeHRes Roadmap and its underlying pillars. The Roadmap consists of 5 interrelated phases: contextual inquiry, value specification, design, operationalization, and summative evaluation [11,24,25]. These phases are connected using formative evaluation cycles. The original Roadmap is supported by pillars that highlight the importance of participatory development, continuous evaluation, and intertwining development and implementation, accounting for changes in the organization of health care because of eHealth, the importance of persuasive design, and the use of advanced methods to assess impact. These pillars are illustrative of the interdisciplinary nature of the Roadmap, which draws from domains such as psychology, persuasive technology, engineering, human-centered design, and business modeling. Over the past years, the original CeHRes Roadmap has been applied to a broad range of contexts and has proven to be a useful tool for researchers and practitioners in the field of eHealth (refer to the study by Kip et al [13] for a partial overview). The broad and extensive use of the Roadmap has led to multiple lessons learned and suggestions for improvements by researchers [26,27]. In addition, since 2011, there have been developments within the interdisciplinary domain of eHealth. For example, participatory development has become more common; there is an increase of well-substantiated design principles and guidelines; knowledge on innovative implementation and evaluation approaches has increased; there is more interdisciplinary collaboration and knowledge; and collaborations between researchers, commercial companies, and health care organizations have resulted in new methods and frameworks [14]. In addition, in the last decade, the terminology has changed and there is a new classification of digital interventions, services, and applications [28]. These new developments and insights call for an update of the CeHRes Roadmap, embracing novel perspectives and advancements. It is important to note that, for consistency reasons, we have decided to use the term “eHealth” instead of digital health (interventions).

In this paper, we present an updated version of the phases and pillars of the CeHRes Roadmap. This update is based on 4 different types of resources. First, we used experiences, lessons learned, and points of improvement that were reported in studies that used parts of the original Roadmap [26,27,29-42]. Second, literature reviews on eHealth development, implementation, and evaluation in general were used [13,14,16,43]. Third, we gathered input from discussions with eHealth researchers from different domains (eg, psychiatry, informal caregiving, planetary health, health psychology, and health sciences) on their experiences with and viewpoints on development, implementation, and evaluation. Fourth, we updated and added new frameworks, models, and methods from the different disciplines that are relevant to the CeHRes Roadmap [11]. More specifically, chapters on psychology and behavior change, technology development models, human-centered design, business modeling, value-based design and requirement engineering, implementation science, and evaluation methods grounded in behavioral sciences were used as foundations for the revisions and updates of the Roadmap [24].

The main objective of this paper is to present the updated version of the CeHRes Roadmap: the CeHRes Roadmap 2.0. The CeHRes Roadmap 2.0 should not be viewed as a new framework or a major revision but as a fine-tuned, improved version of the original Roadmap. In this paper, first, the revised pillars will be presented and explained, including references to the studies on which they are based. Second, the updated phases of the CeHRes Roadmap 2.0 are described, starting with a short explanation of their main objectives, followed by examples of relevant methods and frameworks from different disciplines.

## The CeHRes Roadmap 2.0

**Figure 1:**
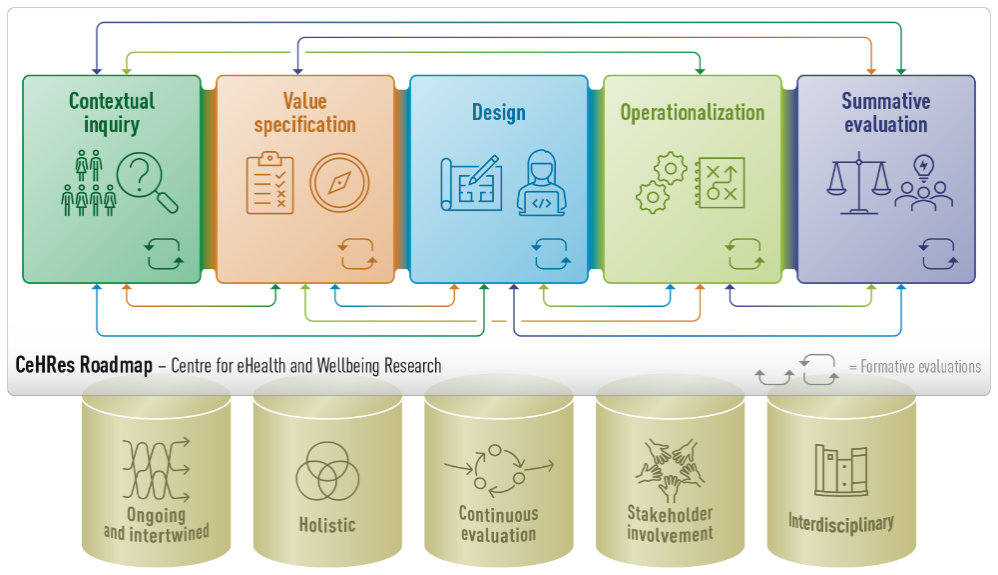
The CeHRes Roadmap 2.0. CeHRes: Centre for eHealth and Wellbeing Research.

In Figure 1, the new visualization of the CeHRes Roadmap 2.0 is provided. In this section, the revised underlying pillars will first be discussed, after which the 5 phases and formative evaluation cycles will be explained.

### The Pillars of the CeHRes Roadmap 2.0

The Roadmap’s pillars provide the foundation upon which it is built. They are the underlying assumptions that are kept in mind throughout all phases of the CeHRes Roadmap 2.0.

#### eHealth Development, Implementation, and Evaluation Processes Are Ongoing and Intertwined Rather Than Phases Containing Separate and Sequential Activities

While it might sound like development, implementation, and evaluation are separate and consecutive stages, this is not the case; they are highly interrelated activities that are all relevant from the start [44]. This also applies to the visualization of many development frameworks; while, for overview purposes, processes or phases are visualized as separate activities, in reality, they are all intertwined [11,24,44,45]. In particular, implementation is seen, still too often, as a postdesign activity, while it plays a role from the start, for example, by identifying potential implementation issues as early as possible to ensure that they can be accounted for during development. In line with this, eHealth development, implementation, and evaluation processes are never really completed; there will always be room for improvement and new questions will keep on arising. However, in practice, this is often difficult to realize because of limited resources, such as time or money.

#### eHealth Development, Implementation, and Evaluation Processes Require a Holistic Approach, Focused on the Fit Between Technology, People, and Context

Regardless of the type or goal of an eHealth intervention, there are interrelationships between the design and content of a technology, the needs and preferences of the people involved, and the context in which it is used [46]. These interrelationships warrant a holistic approach, in which the different elements are all interdependent and part of one whole instead of separate constructs [47]. First, technology and the behavior of people are interrelated. The introduction and sustained use of technology ideally influence the way end users behave, resulting in, for example, better health outcomes or increased well-being. To enable this behavior change, adherence is an important concept, which means that eHealth should be used as intended, which can be viewed as use behavior. Concepts such as engagement—the extent to which someone is involved or occupied with an eHealth technology from an emotional, behavioral, and cognitive perspective—are important to consider as well [48]. Second, the use of eHealth is never isolated, but influences and is influenced by the context in which it is used. The introduction of eHealth creates novel structures and processes for health care delivery, and as a result, an adapted or new ecosystem for health care emerges [49]. Furthermore, the content of a technology can be influenced by contextual factors, such as new treatment protocols.

#### eHealth Development, Implementation, and Evaluation Processes Require a Multimethod, Iterative Approach With Continuous Evaluation Cycles

eHealth development, implementation, and evaluation are not linear processes with consecutive steps; they are iterative, flexible, and dynamic, with constant changes to the process and products [50,51]. This is in line with an agile approach, which is characterized by the division of large tasks into rapid, shorter phases and constant adaptations of plans based on the outcomes of evaluations [52]. To realize this, a multimethod approach is key. Ideally, researchers need to be able to draw from a broad toolkit of qualitative and quantitative research methods and products and select the method that best fits their research objective, the practical demands of the context, and the characteristics, needs, and wishes of the participants [13]. To ensure a coherent approach and to prevent the project team from “getting lost,” there is a need for continuous formative evaluations in which outcomes of activities are critically analyzed, evaluated, and adapted with stakeholders [12]. Such an iterative, agile approach might be challenging and complex and can seem very different from a rigorous scientific approach in which all research activities are meticulously planned. However, such an iterative, agile approach is not a synonym for unstructured or messy; it is a way to shape systematic, structured yet dynamic high-quality research processes that are able to adapt to changes and new insights [52]. Such new insights may lead to the development of new technologies, but this approach is also applicable when new insights lead to re-evaluation and redesign of existing technologies. Consequently, while a broad research plan with potentially suitable methods can definitely be developed, it might be necessary to deviate from this plan based on outcomes at any point during the process or new insights regarding the needs of stakeholders or possibilities of a technology.

#### eHealth Development, Implementation, and Evaluation Processes Require Constant Active Involvement of Stakeholders

To create eHealth that meets the needs and wishes of users and other stakeholders, a participatory approach is recommended, in which stakeholders are actively involved throughout the process, from development to evaluation [30,45,53,54]. Emerging approaches, such as action research and design science highlight that the active involvement of stakeholders goes beyond merely involving end users; stakeholders such as managers, health care insurers, and technology developers are also involved to ensure a holistic approach [40,55,56]. In participatory development, stakeholders can take on different roles, ranging from an informant who mostly provides input on products when invited by researchers to a cocreator who is actively involved in creating ideas and products [57]. While ideally, stakeholders have an active role, in some cases a more passive role might be more suitable for a specific objective. How a stakeholder is involved is not fixed and will differ depending on the phase of the process and the type of activity that is conducted. It is important to ensure that stakeholders are only involved when it truly is of added value. Merely involving end users for the sake of involving them does not benefit anyone involved and takes up the valuable time of participants and researchers [58]. Ideally, stakeholders should be part of an interdisciplinary project team that coordinates the entire eHealth process. Finally, participatory development does not always have to be about creating new technologies, because existing technologies can be redesigned and reused in different contexts as well, which also requires active stakeholder involvement.

#### eHealth Development, Implementation, and Evaluation Processes Are Based on an Interdisciplinary Approach

To capture the complexity of eHealth, an interdisciplinary approach toward research and development is required [12,14]. In such an approach, theories, methods, and models from different disciplines are combined and ideally merged and integrated, resulting in new frameworks, concepts, and models [59]. While there are multiple domains that can be relevant for eHealth, there are some main paradigms that underpin the CeHRes Roadmap 2.0, which are psychology and behavioral sciences, human-centered design, engineering, persuasive technology, implementation science, and business modeling [11,24]. Furthermore, domain-specific theories that are relevant to a specific context or target group often play an important role and should be integrated as well [29]. Finally, the interdisciplinary nature of eHealth is important when composing the project team that coordinates the development, implementation, and evaluation processes. Putting together a team with members (and stakeholders) from different disciplines is deemed essential to ensure that all relevant perspectives are actively involved throughout to prevent tunnel vision [29,60,61].

#### Changes Compared to the Original CeHRes Roadmap

Compared to the first version of the Roadmap, several changes are made to the pillars while retaining the main essence of the original Roadmap. An important change is that the pillars were broadened to better reflect the vision that development, implementation, and evaluation aspects of eHealth are interrelated. For example, in the initial pillars, the term “eHealth development” was used, but in the new version, we broadened this to “eHealth development, implementation, and evaluation processes.” Furthermore, the terminology was adapted to better fit the iterative, interdisciplinary nature of eHealth. For example, the term participatory development was replaced by the term “active involvement of stakeholders.” The phrasing and changes of the updated pillars better capture the essence of the CeHRes Roadmap 2.0.

### The Phases of the CeHRes Roadmap 2.0

#### Overview

Before diving into the phases of the CeHRes Roadmap 2.0, it is important to explain how the Roadmap should be used. It is not a step-by-step guideline but more of a set of underlying assumptions that are relevant to any eHealth process. Strictly speaking, the first 3 phases of the Roadmap 2.0 are related to the development of eHealth, operationalization fits best with implementation, and summative and formative evaluation are most aligned with the Evaluation phase. This distinction into 5 phases is made for visualization and overview purposes, in practice, the first phases will also be relevant for implementation and evaluation efforts and vice versa. In line with this, the visualization of the phases as separate blocks does not in any way imply that the process is sequential; this is also done for overview purposes. In reality, phases overlap and project teams will go back and forth between the different phases, as represented by the arrows in Figure 1. Therefore, it is important to note that the description of the phases of the CeHRes Roadmap 2.0 is not a concrete guideline for creating impactful eHealth interventions. It is meant to serve as a structured overview of principles and methods that can be used by researchers and practitioners to shape their own and unique development, implementation, and evaluation processes. Consequently, methods or frameworks that are not described in this paper can most definitely be used too. A key feature is that they fit within the process and are aligned with relevant research questions. To conclude, the information mentioned subsequently is aimed to support eHealth researchers through inspiration, guidance, and concrete input for guiding eHealth development, implementation, and evaluation processes.

#### Contextual Inquiry

##### Overview

The first step in any eHealth-related project is a thorough investigation of the context in which it will be used. A contextual inquiry is required to paint a picture of the (groups of) people that will come in contact with the technology and their context and to identify the main points of improvement for which an eHealth technology can be of added value. A thorough contextual inquiry assists researchers and developers in keeping a focus on the people and their context from the start of the development process [30,62]. This increases the chances of developing an eHealth technology that provides a solution for relevant issues, fits within a specific context, and is accepted by relevant stakeholders [12,63]. Consequently, the outcomes of a contextual inquiry provide the foundation for all following development, implementation, and evaluation activities. The objectives of the contextual inquiry are described in subsequent sections, including several illustrative examples of suitable methods, frameworks, and concepts that might be used within this phase.

##### To Identify and Gain Insights Into the Roles and Activities of Relevant Stakeholders

Due to the importance of actively involving stakeholders in every phase of the CeHRes Roadmap 2.0, a clear overview has to be provided from the start of the development process. Stakeholder identification can be conducted, using methods such as expert recommendations on stakeholders, snowball sampling with stakeholders, and literature studies [40]. Once an initial overview is created, the roles and tasks of stakeholders have to be analyzed. An example of a useful approach is the stakeholder salience model, in which stakeholders are mapped based on their power, legitimacy, and urgency [64]. On the basis of analyses, key stakeholders who should be involved more intensively throughout the process can be selected.

##### To Analyze the Current Situation Regarding the Involved People, Organizations, Wider Context, and Technologies

A thorough understanding of the current situation is essential to ensure that the project team knows for whom and for what context they are creating eHealth and to specify the focus of the project. Using multiple methods, an overview can be created of the involved people (eg, their daily lives, attitudes, behavior and determinants of behavior), the organizations in which eHealth will be implemented (eg, organizational characteristics, protocols, and domain-specific theories), the wider context (eg, national policies, legislation, and funding possibilities), and information on relevant technologies and technological infrastructures that are or might be used in the intended or other contexts (eg, working mechanisms, experiences with similar technologies, and technological possibilities) [13]. It is important to truly understand the context before going into specific solutions to prevent tunnel vision and to ensure a good fit between the eHealth technology and the involved stakeholders. To achieve this, a context should not be viewed as a single entity but as a complex ecosystem of interrelated platforms, technologies people, organizations, and other stakeholders. Finally, it is important to pay attention to (potential) ethical points of attention from the start of the development process, such as privacy, accessibility, or data ownership.

##### To Identify the Main Points of Improvement for Which an eHealth Technology Could Be a Solution

Merely describing a context is not sufficient for developing eHealth; the project team needs to gain insights into what the points of improvements are in the current situation that can be addressed by a to-be-developed eHealth technology. These points can be related to, for example, efficiency, effectiveness, patient satisfaction, safety, or quality improvement. An important aspect of this activity is to describe the behavior that needs to be changed to improve the situation and identify accompanying determinants that can be targeted by eHealth technology [45]. When identifying and describing points of improvement and accompanying behavior, newly gathered information from stakeholders is combined with scientific sources to increase the robustness of the findings. By integrating methods such as desk research, (systematic) literature reviews, focus groups, interviews, and observations, a complete, multifaceted picture of what has to be changed can be painted [13].

#### Value Specification

##### Overview

In the Value Specification phase, the topics that were identified in the contextual inquiry are narrowed down and translated into values and specific requirements for a to-be-developed eHealth technology. This is again done in close cooperation with the identified stakeholders; however, other stakeholders might also pop up in this phase. Information from the literature is required to be able to incorporate evidence-based behavior change techniques (BCTs) into a technology that facilitates behavior change. Moreover, in this phase, the development team needs to take the first concrete steps toward implementation, among other things, by initiating the development of a business model. While the value specification can be quite complex and time consuming, it is important to put effort into laying the groundwork for the content and design of a technology before diving into its actual creation. This again ensures that the technology is in line with the preferences and characteristics of stakeholders, incorporates evidence-based working mechanisms, and remains in line with the identified points of improvement. The objectives of this phase and examples of accompanying methods are described in subsequent sections.

##### To Formulate and Prioritize Values From All Identified Key Stakeholders and Set Concrete Goals Based on These Values

While there are many definitions of values, in relation to eHealth development, values can be viewed as what stakeholders find important regarding the technology from their personal or professional perspective, also related to, for example, ethics and morality. In other words, values are abstract concepts that represent the ideals or interests of stakeholders and summarize what they find important on an abstract level [38,65]. Values can be related to social, behavioral, cognitive, emotional, economic, or health care issues. Examples are saving costs, increasing access to health care, or improving autonomy [39,65]. Values can be uncovered through (ideally) a combination of multiple methods such as interviews, focus groups, or questionnaires [65]. When collecting data, it is pivotal to carefully consider the intended target audience when finding a fitting way to discuss values. For some target audiences (eg, clients in forensic psychiatry), discussing values in an abstract way (eg, “increase self-control”) can be very complex [66]. In that case, it can help to show and discuss concrete examples of existing or potential technologies [29]. Because different stakeholders are involved, conflicting values are expected to arise, for example, the manager’s perspective might differ from that of the patient. This is not problematic but requires deliberate decision-making and prioritizing by the interdisciplinary project team. To ensure that these more abstract values are translated into a concrete technology, more specific requirements are formulated. Furthermore, to guarantee that values are incorporated throughout the further implementation and evaluation activities, it is useful to formulate accompanying objectives that are formulated in a specific, measurable, achievable, realistic, and timely (SMART) way. These objectives can serve as the basis for evaluation and implementation studies.

##### To Select Initial BCTs and Persuasive Features That Fit With the Values and Needs of the Key Stakeholders

In previous activities, the behavior that needs to be changed was analyzed and the values of the key stakeholders that play a role in doing so were identified. On the basis of that information, fitting behavior change methods can now be selected to increase the chances of actually reaching the intended behavior change and to improve adherence and engagement to the eHealth technology. This is important because changing behavior is highly complicated. To increase the chances of an eHealth technology’s success in actually changing behavior, the chosen strategy should fit the users and what they are trying to achieve. BCTs are derived from behavior change theories from psychology and can be defined as a general technique to influence or create changes in the predictors of specific behavior [67]. Examples of BCT categories are goals and planning, social support, feedback and monitoring, and reward and threat. In addition, persuasive features from the Persuasive Systems Design (PSD) model can be selected [68]. The persuasive features are divided into 4 categories: primary task support, dialogue support, credibility support, and social support. BCTs partly overlap with persuasive techniques because they both target behavior change, but the main difference is that the PSD model is specifically aimed at technology, while BCTs are applicable to any kind of behavior change intervention. Finally, depending on the project, domain-specific theories can also be used to support behavior change. Identifying and integrating behavior change methods in technology is a complex and highly iterative process that will remain relevant throughout the process, but the foundation is laid in the Value Specification phase.

##### To Translate the Values Into Specific Requirements to Describe the to-Be-Developed eHealth Technology

Requirements describe what an eHealth technology should do, what data it should store or retrieve, what content it should display, and what kind of user experience it should provide [38]. There are different types of requirements related to functionality and modality, service, organization, content, usability, and user experience. Particularly due to the increasing importance and the use of (big) data, attention should be paid to data-related topics, such as data ownership, the way data are collected, and how and where to save generated data. Requirements should be related to values; an abstract value is often related to multiple more specific requirements. When formulating requirements, data that were used to formulate values can be used, but new data are often collected as well, for example, through focus groups, interviews, card sorting, and literature reviews [13]. It is also important to integrate the selected potential behavior change mechanisms into requirements to ensure that they are integrated into the technology. A specific product that can help in designing for the end users and facilitating requirement formulation is a persona: a hypothetical archetype of a user presented by means of a short biography with a photo, sociodemographic information, and their values [69,70]. Scenarios can be used to link personas to the technology, because they describe the potential, envisioned use of an eHealth technology by the end users. Scenarios can contain information about how the end user uses a technology in relation to their characteristics and values, what activities are supported by a technology, which steps a user follows, in which context this happens, and what the technology’s main functionalities are [71].

##### To Create the First Version of a Business Model for the eHealth Technology

To prevent a technology from not being used after it has been introduced in practice or not even being introduced at all, it is important to start developing a business model as soon as possible. A business model can be defined as “the rationale of how an organization creates, delivers, and captures value” [72]. A frequently used method to create a business model is the business model canvas, which consists of 9 blocks. These blocks represent the value proposition (eg, the eHealth technology), the organizational activities that are necessary to deliver the eHealth technology to practice, the main customers and users, and financial aspects and cost-benefit ratios. A business model can be filled in using multiple methods, such as focus groups, desk research, and outcomes of earlier development activities [32]. While the initiation of the business model takes place in the value specification, it is adapted throughout the entire process of eHealth development, implementation, and evaluation.

#### Design

##### Overview

While this is not a “hard” transition, the actual eHealth technology is created in the Design phase based on the output of the contextual inquiry and the value specification. This is an extremely dynamic, iterative, creative, and collaborative phase during which there is an active collaboration between end users, other stakeholders, researchers, designers, behavior change experts, content experts, and perhaps even funders. While there are many ways to guide this process, the Double Diamond Model (or framework for innovation) can be used to shape the cycles of iteration between design and testing that are relevant for this phase [73,74]. It is important to note that the final technology is not developed all at once. If that is done, important problems may be missed and arise only after the technology has been introduced in practice, thus wasting a lot of time and effort. Therefore, multiple prototypes, visual representations of the to-be-developed technology, are developed based on the requirements. These prototypes are evaluated by users and experts and are constantly updated in an iterative process. It must be stressed that throughout the prototyping process, outcomes of previous phases (eg, requirements, BCTs, and stakeholder lists) can be adapted or complemented because of new insights, related to, for example, usability, new organizational opportunities, or technological possibilities or impossibilities. To support this process, design principles for digital developments can be used [75]. Furthermore, in the Design phase, insights into barriers that might arise during implementation are gathered, illustrating the interrelatedness between development and implementation.

##### Both Low-Fidelity and High-Fidelity Prototypes of the eHealth Technology Are Developed

Prototypes are visual representations of a technology. They can be crude and more sketch like (low fidelity) or can resemble the to-be-developed technology more closely (high fidelity), but they are in any case based on previously formulated requirements [76]. The first step in the prototyping process is to come up with initial design ideas by means of ideation. These ideas are increasingly specified based on input from stakeholders in formative evaluations throughout the prototyping process. There are several methods to create prototypes, such as paper-based prototyping (eg, sketching), computer-based digital prototyping, storyboards that show how the technology can be used, or 3D prototyping with materials such as cardboard, clay, or 3D printing. Content for the prototypes can be initiated via, for example, literature studies, desk research on existing protocols, card sorting, Delphi studies with experts, and further refined during the design process [13].

##### Persuasive Features, BCTs, and Domain-Specific Theories Are Operationalized and Integrated Into the Design of the eHealth Technology

While an initial overview of potentially useful BCTs and persuasive features was made in the value specification, this set of techniques will most likely be adapted throughout (and even after) the prototyping process, in line with the complexity of behavior change. Furthermore, the techniques have to be operationalized and integrated into prototypes of the design [33]. Integrating persuasive features and BCTs in the design of an eHealth technology requires an iterative and creative approach, for example, using brainstorming and cocreation sessions with stakeholders, involving experts on design and behavior change, and investigating techniques and features in similar technologies. Once these techniques and features are integrated into prototypes, they are evaluated with stakeholders and experts to investigate if they are potentially effective and fit the skills and preferences of end users. While doing so, it is important to also consider checking if the BCTs and persuasive features are integrated successfully into the design according to experts and stakeholders. An example of this is to analyze perceived persuasiveness according to stakeholders [77].

##### Usability Tests of the Prototypes Are Conducted With End Users, Experts, and Other Stakeholders

Prototypes are evaluated with stakeholders to gain insights into their opinions and thus iterate toward the most suitable version of an eHealth technology for a specific target group in a specific context. This can be done by means of usability tests, which can be used to study, for example, how someone interacts with the system, to test the ease of use and user-friendliness of the technology, or to assess whether requirements are correctly translated into the design. There are multiple methods for usability testing [78]. The think-aloud method lets intended end users navigate through a prototype guided by a scenario, a task that is to be dealt with by using the prototype, while verbalizing their thoughts, actions, and ideas. In a heuristic evaluation, design experts assess usability by comparing it to a predefined set of principles such as “recognition rather than recall” or “minimalist design” [79]. In a cognitive walk-through, experts are asked to execute tasks that a user would want to perform [80]. These are just a couple of examples of the multitude of available methods for usability testing.

#### Operationalization

##### Overview

Operationalization refers to the planning and actions for the introduction, dissemination, adoption, and internalization of (a first functioning version of) the technology in the intended context. In this phase, the technology is launched, marketing is set into motion, and organizational working procedures are put into practice. In this phase, the business model is completed and applied to practice, using input from earlier phases as well as by collecting new data. In addition, previously collected data and new research are combined into a concrete implementation plan with specific activities, ideally structured by means of an implementation framework.

##### Complete the Business Model With Input of the Stakeholders and Put It Into Practice

The business model, which was initiated in the Value Specification phase, is made as complete as possible and is rolled out in this phase. Concrete plans on how to act upon the content of the 9 blocks of the business model canvas are created, in close cooperation with stakeholders, using methods such as focus groups, desk research, or interviews [32]. While the business model is made as complete as possible, this does not mean that the model is fixed: chances are that it will be updated constantly, in line with, for example, new insights, policy changes, new customer groups, or new financing structures. Finally, it is important to carefully consider matters related to data in a business model, who owns the data that are collected by technology, and what is the potential value of these data [81].

##### Create an Overview of Implementation Barriers and Facilitators Using Input From the Previous Phases, New Data, and Implementation Frameworks

Because implementation is not a postdesign activity, information on, for example, implementation barriers and facilitators has already been collected throughout the previous activities. In this phase, previous outcomes are combined with newly collected data on implementation factors and strategies to provide an overview of factors to account for when planning, executing, and evaluating implementation processes [43]. The process of planning for implementation can be structured by means of implementation frameworks. A much-used framework is the Consolidated Framework for Implementation Research (CFIR), which incorporates a broad range of factors from existing theories and divides them into 5 domains: innovation, outer setting, inner setting, individuals, and implementation process [82]. Another approach, which is focused on value-based technology in health care, is the Nonadoption, Abandonment, Scale-Up, Spread, and Sustainability (NASSS) framework, consisting of 7 domains: condition, technology, value proposition, adopters, organization, wider system, and embedding and adaptation over time [83]. While there are different implementation frameworks, they all highlight the multilevel nature of implementation and emphasize that it is important to pay attention to a broad range of stakeholders and organizations.

##### Develop a Concrete Implementation Plan Based on the Previously Identified Implementation Factors and Apply It to Practice

On the basis of the previously identified implementation barriers and facilitators, a concrete plan for implementation is comprised, including implementation outcomes and strategies. While there is no step-by-step approach to do this, specific implementation outcomes can be determined to steer and evaluate the process, related to, for example, acceptability, cost, and sustainability [84]. To achieve the objectives, specific implementation strategies are identified, which can be linked to the previously identified barriers and facilitators (ie, factors) [36]. Overviews of implementation strategies can be used to guide this process [85]. Active stakeholder involvement in identifying suitable activities for implementation is important, partly because there is not much evidence-based information on which strategies can be effectively used for overcoming implementation barriers [43,85]. Examples include training health care providers, development of technology-enhanced treatment protocols, or practical support of clinicians in using the intervention [15]. The accompanying materials, such as flyers or training sessions, are ideally cocreated with stakeholders.

#### Summative Evaluation

##### Overview

While summative evaluation is visualized as the final part of the CeHRes Roadmap 2.0, it is not a postimplementation and development activity but an iterative, multimethod process that is highly intertwined with and based on activities and outcomes from development and implementation processes [16]. In evaluation processes, researchers investigate to what extent the eHealth technology succeeds in addressing the objectives formulated in previous phases. For overview purposes, a rough distinction can be made between 3 aspects of eHealth evaluation, although they overlap. The first aspect is the evaluation of the *impact* of eHealth on its users, stakeholders, and their context, mostly aimed at assessing its benefits and effects on different types of outcomes (eg, clinical, organizational, and behavioral). The second aspect is related to assessing the *uptake* of eHealth to gain insight into how, by whom, and when a technology was used. The third element is related to the evaluation of *working mechanisms* to gain insight into why an eHealth technology was effective or not and why it was used. By combining these types of evaluations, insights can be gained into if, why, how, for whom, and when eHealth works, and points of improvement for its design and implementation can be identified. Several frameworks can be used to shape these complex, multifaceted evaluation processes. Examples are the Multiphase Optimization Strategy (MOST) framework, which consists of 3 phases: preparation, optimization, and evaluation [86]; Process evaluation, in which attention is paid to the relationship between context, implementation, and mechanisms of impact, and their influence on the outcomes of an intervention [87]; or realist evaluations, in which information is collected to build Context-Mechanism-Outcome configurations to explain how a specific context triggers specific mechanisms that lead to particular outcomes [88]. These approaches can all be used for the evaluation of complex interventions and account for the role of the context, the technology, and people when evaluating an eHealth technology.

##### Determine the Impact of the eHealth Technology on the Users, Other Stakeholders, and Their Context

To assess the impact of eHealth, it is important to translate the previously formulated values and accompanying objectives into research questions. These can relate to, for example, clinical values, lifestyle behaviors, quality of care, efficiency, or other types of benefits. To paint a comprehensive picture of the influence of eHealth on the users and contexts, a multi- or mixed methods approach can be used. The choice of a method depends on the research questions and characteristics of the target group and context. Some examples of methods that can be used are randomized controlled trials (RCTs), interviews or focus groups, single-case experimental designs, or health technology assessments. When choosing what method to use, it is important to thoroughly consider the context in which a technology is used and keep an eye on the limitations of a specific method [89].

##### Analyze the Uptake of the eHealth Technology in Terms of Adoption or Use of the Technology by Its Intended Users

Evaluations of uptake are used to gain insight into by whom, how often, when, and how an eHealth technology is used. This type of evaluation can be used to answer questions about, for example, how often users logged in, how often a technology was used by different organizations, and what features of it were used or not and in what order. While there are multiple ways to answer these questions, log data analysis is a commonly used method [37,90]. By gaining more insights into the use of technology and by connecting it to outcomes, the black box of eHealth can be made more transparent. To illustrate, if an RCT shows no significant effects for an intervention, evaluation of uptake can be used to explain if this is because the intervention itself does not work or if (an element of) the intervention was simply not used. To incorporate these types of data in evaluations, it is important to carefully determine how data are collected and stored from the start of the development process [90]. Furthermore, this type of evaluation also provides points of improvement for implementation in practice or the design of an eHealth technology.

##### Investigating Relevant Working Mechanisms That Explain Why an eHealth Intervention is Effective or Not for Its Users

Understanding why an eHealth technology achieves a certain effect, rather than “just” evaluating *if* it achieves an effect, can provide generalizable insights into how health behavior change can be achieved via an intervention [87]. This knowledge can be used to make future interventions more effective or targeted. There are many different concepts that can be used to gain insights into working mechanisms, for example, related to sociodemographic characteristics of its users, such as age or personality, but ideally, these concepts are more overarching. Examples are previously mentioned BCTs and persuasive features or broader concepts such as adherence, the extent to which a technology is used as intended [91], and engagement, the extent to which someone is involved or occupied with an eHealth technology from an emotional, behavioral, and cognitive perspective [48]. There are several methods that can be used to gain more insights into the working mechanisms of eHealth technologies, such as mixed methods approaches with in-depth qualitative research or (fractional) factorial designs in which different versions of an intervention (eg, one with and one without a specific BCT or persuasive feature) are evaluated. It is important to note that identifying working mechanisms is extremely difficult because there are many combinations of factors that influence the effectiveness or ineffectiveness of a technology, such as characteristics of a specific context, the technology itself, or a broad range of individual factors.

#### Formative Evaluation

##### Overview

While the previously described phases are visualized as blocks in the CeHRes Roadmap 2.0, formative evaluation is represented by the arrows within and between these blocks, also see Figure 1. This is in line with the nature of formative evaluation; it is a principle that is intertwined in and part of all phases of the Roadmap. The basic assumption of formative evaluation is that information on how to improve the process and the eHealth technology itself is continuously collected. This information assists the project team in ensuring that there is a constant focus on the context and people involved and can be referred to as “creating by evaluating.” A distinction can be made in formative evaluation within activities in a phase, related to ensuring that outcomes are aligned with perspectives of stakeholders and contexts, and between activities, referring to ensuring consistency between outcomes of different activities and the different phases.

##### Using Methods to Gather Information From the Stakeholders and Context to Continuously Account for Their Perspectives Within the Activities and Phases of the CeHRes Roadmap 2.0

Formative evaluation within activities and phases emphasizes the importance of gathering stakeholder input and verifying outcomes with them to ensure that products fit the involved people and context. For example, requirements are verified by stakeholders in the Value Specification phase, usability tests of prototypes are executed in the Design phase, and stakeholder input is gathered to create and finetune a business model in the Operationalization phase.

##### Checking Whether the Outcomes of Previous Phases of the CeHRes Roadmap 2.0 Have Been Accounted for in the Current Phase and Ensuring That the Outcomes of all Phases Form a Coherent Whole

Formative evaluations between phases ensure consistency and a clear relationship between the outcomes of all phases. For example, in the Design phase, the project team makes sure that the designed technology incorporates the requirements and addresses the values. This can be done through focus groups with stakeholders or project team meetings. There are no specific methods that must be used, but thorough, transparent documentation of activities and outcomes is essential. If no clear documentation is available, it is hard to retrieve what the main outcomes of previous phases were and what the grounds for specific decisions were.

#### Changes Compared to the Original CeHRes Roadmap

On the basis of the revised pillars and researchers’ experiences, adaptations were made to the objectives, frameworks, and methods within the phases of the Roadmap: contextual inquiry, value specification, design, operationalization, summative evaluation, and formative evaluation. Generally, the objectives of the phases were fine tuned and clarified throughout. Notable modifications include that first, more attention is paid to creating a theoretical foundation for eHealth technologies [14,92,93]. Among other things, the importance of the use of evidence-based BCTs, integration of domain-specific theories, and connection to existing treatment protocols is emphasized. Second, the role of implementation is further elaborated on highlighting the importance of integrating technology in practice to create impact [14,43,89]. For example, implementation frameworks are now described in the Operationalization phase, and the importance of implementation during development and evaluation is further explained. Third, more attention is paid to novel evaluation methods and approaches [16,94]. To illustrate, attention is paid to methods for impact and uptake evaluations, but also to methods for identifying underlying working mechanisms. In addition to that, evaluation models that can be used to shape multimethod, iterative processes are now introduced.

## Discussion

### Overview

In this paper, the CeHRes Roadmap 2.0 was introduced and substantiated based on experiences with the Roadmap and new developments within disciplines that underpin it. The CeHRes Roadmap 2.0 consists of the same phases as the first version, but their objectives have been adapted, reflecting the increased emphasis on BCT, implementation, and evaluation as a process [14]. The pillars have been revised to better reflect new insights regarding the ongoing and intertwined, holistic, iterative, participatory, and interdisciplinary nature of eHealth development, implementation, and evaluation processes. Consequently, while the foundation of the CeHRes Roadmap 2.0 has remained the same, it has been thoroughly updated to better reflect needs from practice and research. The CeHRes Roadmap 2.0 specifically highlights the importance of interdisciplinary collaboration with other researchers and stakeholders from practice; the need for agile, iterative processes; and the importance of accounting for implementation and evaluation from the start of any development process.

### Comparison With Prior Work

#### The Importance of Flexibility

When using any framework for eHealth development, it is important to use it as a set of guidelines to guide unique processes as opposed to a static step-by-step guideline with predetermined methods and frameworks. Ideally, the choice of methods used depends on the phase in the process, characteristics and skills of participants, and practical preconditions such as time and money and not on the methods which the involved researchers happen to be familiar with [13,29,95]. Such a flexible approach is particularly relevant for the CeHRes Roadmap 2.0 due to its holistic nature, in which context, people, and technology are intertwined, highlighting the importance of matching methods and frameworks to the specific technology, people, and organization that are involved [11]. In addition, the iterative nature of eHealth development in general implies that the choice of a method also depends on the outcomes of a previous phase. This means that while the broad outlines of an eHealth project can be planned, the specific methods and their outcomes cannot be planned in detail in advance [1]. To illustrate, at the start of a development process, it is often not yet known what the specific requirements of stakeholders are and what the to-be-developed eHealth technology will look like. Consequently, there also cannot be a concrete plan for its implementation and evaluation in practice. This approach requires flexibility from researchers and perhaps also a change in mindset, shifting from meticulously planned study designs to a more agile, adaptive approach with multiple interrelated methods [50,51]. Of course, to ensure transparency, replicability, and robustness, these smaller steps should be documented well, for example, by publishing protocols in advance on open-access platforms. This mindset shift toward agile science is also relevant for many organizations and funding agencies that often desire or require a specific research plan for multiple years with detailed descriptions of products or outcomes. Fortunately, this agile approach to research is gaining ground, and with this revised version of the Roadmap, we hope to contribute to this much-needed shift. To conclude, while for overview purposes, eHealth frameworks such as the Roadmap are visualized as a sequential set of phases, and specific examples of methods and frameworks are provided, this does not mean that this process should be followed strictly. The exact way of shaping eHealth development, implementation, and evaluation processes is the task of an interdisciplinary project team and requires constant weighing and re-evaluating the aims and limitations of a specific project.

#### Interdisciplinary Collaboration

To tackle wicked societal problems, researchers from different disciplines have to join forces. Consequently, multi- or even interdisciplinary approaches are necessary to address complex, multifaceted challenges, such as developing, implementing, and evaluating effective and efficient eHealth technologies to address problems in health care. eHealth research is often multidisciplinary, in which input is provided by different disciplines, but this is done rather independently from each other, that is, there is not much integration of different methods, approaches, or theories, and people keep working within the boundaries of their discipline. However, to capture the complexity of eHealth, an interdisciplinary approach is advocated [12,14,22,41], in which input from different disciplines is actively integrated, creating a synergy that goes beyond the sum of the individual, disciplinary contributions [59]. Due to the integration of different disciplines, the CeHRes Roadmap 2.0 can be considered an interdisciplinary framework [11]. An example is the combination of BCTs (psychology), persuasive features (persuasive design), and their integration into requirements (engineering and human-centered design) within the Value Specification phase. To further complicate the already intricate task of interdisciplinary work, many researchers also advocate active collaboration with experts by experience such as patients and health care professionals, and other stakeholders such as technology developers or policy makers [12,14,44]. Consequently, people with different backgrounds and points of view have to actively work together, combine their different perspectives, and be willing to look beyond the borders of their paradigm. An important precondition for this is that eHealth researchers have to be able to speak the language of other disciplines [96]. For example, while a psychologist does not have to fully master programming skills, they do have to understand its basics to effectively communicate with developers and prevent misunderstandings. On top of that, eHealth researchers also have to be able to adapt their communication to fit the specific skills of specific groups of stakeholders, such as vulnerable, hard-to-involve patient populations [66]. It is also recommended that researchers be acquainted with and sometimes even skilled in using different types of qualitative and quantitative research methods from different disciplines to be able to select the most appropriate method or at least understand the rationale behind a decision for a method. The domain of eHealth—including the Roadmap—is already shifting from interdisciplinary to transdisciplinary collaboration, where traditional disciplinary boundaries fade even more, and entirely new approaches are developed. This transdisciplinary process also involves close collaboration with community partners on a shared, “wicked” problem [97]. Particularly when working in a transdisciplinary way, researchers should have the ability to look beyond the boundaries of their discipline and really collaborate with societal stakeholders to contribute to a collaboration that is more than the sum of its parts.

#### Continuous Improvement of the CeHRes Roadmap

eHealth technologies and their development, implementation, and evaluation are never really finished. The same goes for eHealth frameworks such as the CeHRes Roadmap 2.0. New insights that urge their further adaptation will arise during the upcoming years not only because of developments of theories and models that are part of the interdisciplinary Roadmap and the fast pace of technological innovations that open up new arrays of opportunities and challenges but also because the Roadmap’s application to practice will result in points of further improvement. To constantly improve frameworks such as the CeHRes Roadmap 2.0 in a systematic way, there is a need for studies in which multimethod development, implementation, and evaluation processes are presented and reflected upon [13,95]. Examples are reflections on the application and suitability of specific combinations of research methods or lessons learned about the operationalization of specific frameworks in a specific health care setting [29]. However, to date, most studies related to eHealth development, implementation, or evaluation only describe the outcomes of a single study, for example, an RCT. Outcomes from smaller studies, such as single-case studies, pilot studies, or qualitative research, that were used to set up the larger study are not reported, either because these types of studies were not performed at all or because authors (or journals) do not feel that publishing them is relevant or important [13,14,98]. However, just as is the case for eHealth technologies, input from stakeholders with different backgrounds is required to constantly improve the Roadmap to ensure that it fits the needs and wishes of its end users. Consequently, there is a need for more multimethod studies that report on the application of (large parts of) eHealth frameworks, critically reflect on their application, and provide points of improvement. To achieve this, future research can focus on creating a standardized way of reporting on multimethod eHealth processes [13,14] comparable to guidelines, for example, systematic reviews [99], RCTs [100], or qualitative research [101], of which an overview is provided by the EQUATOR Network.

### Limitations

Despite its usefulness for eHealth development, implementation, and evaluation, the CeHRes Roadmap 2.0 has some limitations. First, the update was not based on a new systematic review of the literature. Yet, the initial version of the Roadmap was based on a comprehensive review of relevant models and frameworks, and a preliminary exploration of the literature indicated that a repetition of this review would likely not lead to any major new insights. Furthermore, although no new review was conducted, we did ground the CeHRes Roadmap 2.0 on reviews about important elements of the Roadmap, for example, on participatory development [13], implementation [43], evaluation [16], or eHealth frameworks in general [14]. In addition, models and frameworks that were already part of the Roadmap were updated based on new insights, resulting in a comprehensive updated version that covers the most important points of improvement. Regardless of these efforts, the fact remains that, as mentioned before, new suggestions and points of improvement will arise, warranting multiple future recurring updates of the Roadmap.

Second, currently, the Roadmap has mostly been used for the development, evaluation, and implementation of digital health interventions for persons and health care providers. Therefore, the revisions within this second edition are also based mostly on experiences with the development processes of these types of eHealth technologies. Although in principle, the Roadmap 2.0 is also applicable to, for example, the development of digital health interventions for health management and support personnel, and for data services, its value for these areas should be studied more thoroughly.

Another point of attention is that using the CeHRes Roadmap 2.0 does not guarantee the development of effective eHealth interventions that will be widely used in practice. Development, implementation, and evaluation are all extremely complex processes, and while the Roadmap can help to improve them and provide guidance, failures or undesired outcomes might (and will) still occur. In line with this, it is neither possible nor feasible to investigate if using the Roadmap results in better eHealth technologies than using other frameworks such as the person-based approach [53], the Accelerated Creation-to-Sustainment model [44], intervention mapping [45,92], or agile science approaches [50,51]. A comparison of frameworks would require the evaluation of multiple parallel, similar processes. Such similarities are practically impossible to achieve because of the very nature of the CeHRes Roadmap 2.0 and related frameworks and their application to practice, which are all highly dependent on research teams, contexts, objectives, and even periods in time. This makes reliable cross comparisons virtually impossible. Therefore, gaining insights into the added value of frameworks like the Roadmap mostly requires sharing experiences with and lessons learned from its application, which again emphasizes the importance of systematically reporting and reflecting on development, implementation, and evaluation processes, in line with reporting guidelines [13,14,31].

### Conclusions

This study addresses the need for an up-to-date version of the CeHRes Roadmap using the most recent insights and most relevant approaches and models. The changes made in the updated version of the CeHRes Roadmap 2.0 compared to its original version address the need for more attention to behavior change theory in eHealth design; the increased emphasis on implementation; and the importance of iterative, multimethod evaluation processes. To ensure that eHealth frameworks are optimally aligned with experiences and best practices from researchers, there is a need for more studies that apply and reflect on the application of these frameworks because, just as any eHealth technology the CeHRes Roadmap 2.0 has to be constantly improved based on the input of its users.

## Abbreviations

BCT: behavior change technique
CeHRes: Centre for eHealth and Wellbeing Research
CFIR: Consolidated Framework for Implementation Research
MOST: Multiphase Optimization Strategy
NASSS: Nonadoption, Abandonment, Scale-Up, Spread, and Sustainability
PSD: Persuasive Systems Design
RCT: randomized controlled trial
SMART: specific, measurable, achievable, realistic, and timely
